# Cyclic Lipopeptide Biosynthetic Genes and Products, and Inhibitory Activity of Plant-Associated *Bacillus* against Phytopathogenic Bacteria

**DOI:** 10.1371/journal.pone.0127738

**Published:** 2015-05-29

**Authors:** Isabel Mora, Jordi Cabrefiga, Emilio Montesinos

**Affiliations:** Laboratory of Plant Pathology, Institute of Food and Agricultural Technology-XaRTA-CIDSAV, University of Girona, Campus Montilivi, 17071, Girona, Spain; International Centre for Genetic Engineering & Biotechnology, ITALY

## Abstract

The antibacterial activity against bacterial plant pathogens and its relationships with the presence of the cyclic lipopeptide (cLP) biosynthetic genes *ituC* (iturin), *bmyB* (bacillomycin), *fenD* (fengycin) and *srfAA* (surfactin), and their corresponding antimicrobial peptide products have been studied in a collection of 64 strains of *Bacillus* spp. isolated from plant environments. The most frequent antimicrobial peptide (AMP) genes were *bmyB*, *srfAA *and *fenD * (34-50% of isolates). Most isolates (98.4%) produced surfactin isoforms, 90.6% iturins and 79.7% fengycins. The antibacterial activity was very frequent and generally intense among the collection of strains because 75% of the isolates were active against at least 6 of the 8 bacterial plant pathogens tested. Hierarchical and correspondence analysis confirmed the presence of two clearly differentiated groups. One group consisted of *Bacillus* strains that showed a strong antibacterial activity, presented several cLPs genes and produced several isoforms of cLPs simultaneously, mainly composed of *B*. *subtilis *and *B*. *amyloliquefaciens*, although the last one was exclusive to this group. Another group was characterized by strains with very low or none antibacterial activity, that showed one or none of the cLP genes and produced a few or none of the corresponding cLPs, and was the most heterogenous group including *B*. *subtilis*, *B*. *licheniformis*, *B*. *megaterium*, *B*. *pumilus*, *B*. *cereus *and *B*. *thuringiensis*, although the last two were exclusive to this group. This work demonstrated that the antagonistic capacity of plant-associated *Bacillus *against plant pathogenic bacteria is related to the presence of cLP genes and to the production of the corresponding cLPs, and it is mainly associated to the species *B*. *subtilis *and *B*. *amyloliquefaciens*. Our findings would help to increase the yield and efficiency of screening methods to obtain candidate strains to biocontrol agents with a mechanism of action relaying on the production of antimicrobial cLPs.

## Introduction

*Bacillus* is one of the most well-studied and characterized genus of Gram-positive bacteria because of the interest in the agronomic, pharmaceutical and industrial fields due to its ability to form endospores, and to produce a vast amount of metabolites with biothecnologial applications. *Bacillus* species have several characteristics that make them suitable for agricultural applications as good candidates for biocontrol agents (BCAs) of plant pathogens. This is because its ability to produce a wide variety of bioactive compounds [1], several of them with antimicrobial activity [2–5], surface-active properties [6,7], and induction of plant defense responses and growth promotion [8–11]. The characterization of genomes of several *Bacillus* strains have helped to increase the knowledge of genes involved in the synthesis and regulation of these compounds [12–15].

Several studies have been performed in an attempt to elucidate the mechanisms involved in biological control of plant diseases by *Bacillus* species, and antibiosis has been revealed as an important mechanism in most cases [1,3,5,16]. AMPs represent the predominant class of compounds implicated in these cases, being cLPs of the surfactin, iturin and fengycin families the most studied [1,3].

The occurrence of cLPs mediated antibiosis has led to the suggestion that non-ribosomal peptide synthetase genes may be used as markers for identifying and selecting novel biocontrol agents from environmental samples [17]. Primers for PCR analysis have been reported for some antimicrobial biosynthetic genes in *Bacillus* strains antagonistic to fungal and bacterial plant pathogens [18–22]. In certain *Bacillus* strains it was described that antifungal activity was related to the multi-production of AMPs [23], or with the presence of AMP biosynthetic genes simultaneously [20–22, 24]. Also, in the strain *B*. *amyloliquefaciens* GA1, the presence of eight gene clusters directing the synthesis of bioactive peptides and polyketides has been related with the production of the corresponding products [25]. In addition, strains producing multiple AMPs were reported as efficient in the control of *Fusarium* wilt of chickpea [26] and of powdery mildew of cucurbits [27,28]. However, all the above mentioned works were done with one or few strains or with restricted collections of isolates that were selected previously as *in vitro* antagonists or as active in biological control of given fungal plant pathogens. Thus, there is a lack of sufficient information on the relationships of antimicrobial activity with the production of cLPs and the presence of the corresponding biosynthetic genes within natural populations of plant-associated *Bacillus*. Finding this kind of relationships would help to increase the performance of screening methods to obtain candidate strains to biocontrol agents with a mechanism of action relaying on the production of antimicrobial cLPs.

The aim of the present work was to find relationships between cLP genes, products and antibacterial activity that can be useful to increase the efficiency of screening methods to obtain new *Bacillus* strains to be used as biocontrol agents. The study was performed with a collection of plant-associated *Bacillus* strains obtained from a wide origin (diferent plant parts, host plants and locations), and consisted of: (1) an analysis of the distribution of four cLP biosynthetic genes, the cLPs produced, and the antibacterial activity against eight representative plant pathogenic bacteria, and (2) a statistical analysis of the relationships between the antibacterial activity and the production of cLP products and the presence of the corresponding genes.

## Materials and Methods

### Bacterial strains, growth conditions and DNA extraction

A large collection of *Bacillus* isolates was build-up from samples of 183 field sites consisting of plants grown on roadsides, field crop sorroundings, or public forest. Plants were not included in a protection zone, and correspond to not protected or not endangered species. The isolates were confirmed to pertain to *Bacillus* genus based on amplification of the 16S rDNA gene [22]. The material sampled consisted of partial plant parts (leaf, flower, roots), not affecting the survival of the individual. This collection was composed of 184 isolates from 35 cultived and wild-type plant species as described previously [22]. However, this large number of isolates was difficult to be managed for a complete analysis of cLPs. Thus, a sub-sample of 64 isolates was selected at random from the original strain database (Table 1). The *Bacillus* collection was characterized along with two reference *Bacillus* strains, *B*. *amyloliquefaciens* QST713 (Bayer Cropscience, Germany) and FZB42 (Abitep, Berlin, Germany). As targets for the antibacterial activity of the *Bacillus* strains, the following bacterial plant pathogens were used: *Erwinia amylovora* PMV6076 (INRA, National Institute of Agronomic Research, France), *Pseudomonas syringae* pv. syringae EPS94 (CIDSAV, University of Girona, Spain), *Clavibacter michiganensis* sbsp. *michiganensis* CECT790, *Pectobacterium carotovorum* CECT225, *Ralstonia solanacearum* CECT125, *Rhizobium radiobacter* CECT472 (Spanish Type Culture Collection, Valencia, Spain), *Xanthomonas arboricola* pv. fragariae CFBP3549 and *Xanthomonas axonopodis* pv. vesicatoria CFBP327 (CFBP, French Collection of Plant Pathogenic Bacteria, UMR PaVé-INRA, France).

**Table 1:** Location, type and host species of the samples used for isolation of *Bacillus* in the present study.

Sample location	Zone	Latitude	Longitude	Sample type	Number of samples	Number of isolates	Isolates
Girona	Canet de la tallada (Baix Empordà)	42° 03’ N	1° 40’ E	Aerial plant part	3	4	A43, A44, A45, A46
	Estartit (Baix Empordà)	42° 30' N	3° 11' E	Aerial plant part	13	19	A4, A5, A7, A11, A14, A16, A17, A18, A19, A22, A23, A24, A25, A26, A29, A30, A31, A32, A62
				Rhizosphere	8	17	A1, A2, A3, A6, A8, A9, A10, A12, A13, A15, A20, A21, A27, A28, A44, A59, A60, A61
	Mas Badia (Baix Empordà)	42° 14’ N	2° 51’ E	Aerial plant part	4	6	A33, A34, A35, A36, A37, A38
	Torroella de Montgrí (Baix Empordà)	42° 02' N	3° 07' E	Aerial plant part	1	1	A63
				Rhizosphere	1	1	A64
Lleida	Vallferrera (Pallars Sobirà)	42° 35' N	1° 19' E	Aerial plant part	1	1	A41
Menorca	Es Mercadal	39° 59' N	4° 05' E	Aerial plant part	1	1	A42
	Cap de Cavalleria	40° 05' N	4° 08' E	Aerial plant part	3	3	A54, A56, A58
				Rhizosphere	2	2	A55, A57
Tarragona	Les Borges del Camp (Baix Camp)	41° 10' N	1° 01' E	Aerial plant part	7	7	A47, A48, A49, A50, A51, A52, A53
Valencia	Riola (Ribera Baixa)	39° 11' N	0° 20' W	Aerial plant part	1	1	A40
Zaragoza	La Almunia de Doña Godina (Valdejalón)	41° 30' N	1° 24' W	Aerial plant part	1	1	A39

Bacterial strains were cultured in LB agar at 28°C for 24 h. Concentrations of cell suspensions were obtained using a standard curve relating cell concentration measured by CFU, to optical density (OD) at 620 nm (Aurius 2000 series, CE2021, CECIL Instrumentls Limited, Cambridge) (10^8^ CFU ml^-1^ ranging from 0.15 to 0.4 OD depending on the bacteria). For DNA extraction, reference strains and field isolates were cultured in LB agar at 28°C for 24 h. A cell suspension was prepared at 10^8^ CFU ml^-1^ and DNA extractions were carried out using QIAamp DNA Mini Kit (Qiagen Iberia S.L., Madrid, Spain).

### Isolates identification

Identification at species level of the *Bacillus* strains was conducted by *16S rDNA* sequence comparison of the PCR-amplified products using primers 16SEQF1 (5’-GCGGCGTGCCTAATACAT-3’) and 16SEQR3 (5’-TAAGGTTCTTCGCGTTGCTT-3’) under the following conditions. For the PCR assay, the reaction volume was 100 μl and included 1 X PCR buffer, 1.5 mM MgCl_2_, 0.2 mM dNTP (Invitrogen Technologies), 0.2 μM of each primer set, 1.0 U of Taq DNA polymerase (Invitrogen) and 5 μl (aprox. 100 ng) of genomic DNA. The following cycling conditions were used to amplify all targets: 5 min at 94 °C, 35 cycles of 30 sec at 94°C, 30 sec at 52°C, and 1 min at 72°C. A final extension step of 7 min at 72°C was followed by a 4°C hold step. Amplifications were carried out in a T3000 thermocycler (Biometra, Germany). The amplification products were separated on a 1.8% agarose gel in 1X Tris-acetate EDTA (TAE) for 45 min at 90 V and viewed after staining with ethidium bromide. Gel images were captured with ChemiDoc XRS+ System (Bio-Rad, USA). PCR products wer purified by means QIAquick PCR Purification Kit (QIAGEN, Valencia, CA, USA). Base sequences were determined using Macrogen's sequencing service (Macrogen Europe, Amsterdam, Netherlands). The 16s rDNA sequences of the strains were compared to known sequences in the DNA sequence database at the National Center for Biotechnology Information (NCBI), and an additional BLAST search was performed. For sequence comparisons, a sequence datamatrix of the *16S rDNA* genes from 73 *Bacillus* was constructed, including 57 isolates and 16 nucleotide sequences of *Bacillus* reference strains from the GenBank database. These sequences were aligned using Mega 6 [29]. The variable and incomplete sites at both the 5′ and 3′ ends of the *16S rDNA* gene sequences were excluded from the alignment. Phylogenetic trees were inferred using the neighbor-joining (NJ) algorithm with the maximum composite likelihood model using MEGA 6.0.

### cLP genes detection

*Bacillus* isolates and reference strains were characterized using four antimicrobial peptide biosynthetic genes chosen within the coding regions of *ituC* (iturin A synthetase C), *bmyB* (bacillomycin L synthetase B), *fenD* (fengycin synthetase) and *srfAA* (surfactin synthetase subunit 1), using the specific primers ITUCF (5’-GGCTGCTGCAGATGCTTTAT) and ITUCR (5’-TCGCAGATAATCGCAGTGAG), BMYBF (5’-GAATCCCGTTGTTCTCCAAA) and BMYBR (5’-GCGGGTATTGAATGCTTGTT), FENDF (5’-GGCCCGTTCTCTAAATCCAT) and FENDR (5’-GTCATGCTGACGAGAGCAAA), and SRFAF (5’-TCGGGACAGGAAGACATCAT) and SRFAR (5’-CCACTCAAACGGATAATCCTGA). PCR assays were carried out at least three times for each isolate as previously reported [22].

### Analysis of cLPs

The ability of the *Bacillus* isolates of the collection and the reference strains (QST713 and FZB42) to produce cLPs was analyzed. Antimicrobial peptides were extracted, purified and identified by the combination of two techniques departing from culture supernatants. 2 ml of *Bacillus* suspension adjusted to 10^8^ CFU ml^-1^ were inoculated in 100 ml of LB broth medium contained in 500 ml Erlenmeyer flasks. Cultures were incubated at 28°C for 48 h and 180 rpm in an orbital shaker incubator. Cell-free supernatants were obtained by centrifugation at 12000 × g for 15 min and filtered through cellulose acetate syringe filters (0.2 μm pore size). Isoamyl alcohol was added 50% (v/v) to the supernatant and the mixture was vigorously shacked. The organic phase was separated by centrifugation at 12000 × g for 5 min at room temperature. The organic phase was collected and evaporated under vacuum (Scan Speed 40 Teflon, AAPPTEC, LCC, Louisville, USA). Dried concentrate were stored at -80°C.

The corresponding organic phase was analyzed by high-performance liquid chromatography (HPLC) and characteristic elution peaks were separated and collected by preparative HPLC. The analytical HPLC was performed on an Agilent 1200 HPLC system (Agilent Technologies, Palo Alto, CA) using a reverse-phase Kinetex XB-C18 column (Phenomenex, Madrid, Spain). The elution was carried out by the following conditions: linear gradient 2–50% of acetonitrile containing 0.085% TFA for 18 min, linear gradient 50–100% of acetonitrile for 4 min, at constant flow rate of 1.85 ml/min. The eluted compounds were detected by monitoring absorbance at 220nm. The preparative HPLC was performed using a Spherisorb ODS2 C-18 column (Waters Corporation, Milford, USA). The elution was carried out by the following conditions: linear gradient 2–40% of acetonitrile containing 0.085% TFA for 4 min, linear gradient 40–50% of acetonitrile for 16 min, linear gradient 50–100% of acetonitrile for 3 min, at constant flow rate of 6 ml/min. The eluted compounds were detected by monitoring absorbance at 220nm. Fractions were lyophilized and stored at -80°C.

Components were identified according to their mass using matrix assisted laser desorption/ionization time-of-flight (MALDI-TOF) and electrospray ionization mass spectrometry (ESI-MS). Fractions were dissolved in distilled water supplemented with trifluoroacetic acid (TFA) 0.085%, until a final concentration tenfold concentrated with respect to the original extract. MALDI-TOF analysis was performed in Bruker Daltonics Ultraflex equipment (Bruker Daltonics, Bruker Corporation, Billerica, USA). Then, 1 μl of each sample was mixed with 1 μl of matrix solution, which consisted of a saturated solution of α-cyano-4-hydroxycinnamic acid in 30% aqueous acetonitrile containing 0.1% TFA (v/v). 1 μl of the mixture was deposited in spots on a MALDI plate and dried with the help of air flow cabin. The acceleration and reflector voltages were 20–28.5 and 23–30 kV, respectively. Mass spectra were taken and recorded by using a nitrogen laser (337 nm, repetition rate 20 Hz) for desorption and ionization (acquisition mass range: 500–3000 m/z, low mass gate: 200 m/z). All experiments were carried out with the reflector positive ion mode. Data were analyzed with the software flexAnalysis v 2.0 (Bruker Daltonics). ESI-MS analyses were done in an Esquire 6000 ESI ion Trap LC/MS (Bruker Daltonics, Bruker Corporation, Billerica, USA) equipped with an electrospray ion source. In parallel, samples of 5 μl, consisting in purified elution peaks were introduced into the mass spectrometer ion source directly through a HPLC autosampler. The mobile phase flow (100 μl/min of 80:20 v/v Acetonitrile/H_2_O) was delivered by a 1200 Series HPLC pump (Agilent). Nitrogen was employed as both drying and nebulizing gas.

### Antibacterial activity assays

The ability of the *Bacillus* isolates and reference strains (QST713 and FZB42) to inhibit eight bacterial plant pathogens (*Erwinia amylovora* PMV6076, *Pseudomonas syringae* pv. syringae EPS94, *Xanthomonas arboricola* pv. fragariae CFBP3549, *X*. *axonopodis* pv. vesicatoria CFBP3275, *Rhizobium radiobacter* CECT472 (syn. *Agrobacterium tumefaciens*), *Ralstonia solanacearum* CECT125, *Clavibacter michiganensis sbsp*. *michiganensis* CECT790 and *Pectobacterium carotovorum sbsp*. *carotovorum* CECT225) was determined using an *in vitro* agar plate assay. Two different growth media, LB agar and Nutrient agar (NA), were used independently. The assay was based on the overly method and agar overlays were prepared by mixing 4.5 ml of melted agar (0.7% at 45°C) and 0.5 ml of each bacterial pathogen (10^8^ CFU ml^-1^) and covering the surface of a LB or NA agar plate. *Bacillus* strains grown overnight on LB agar plates at 28°C were transferred to the surface of the overlay with toothpicks. Three independent replicates of each *Bacillus* isolate were used to assess the growth inhibition of each pathogen. Plates were incubated at 25°C and growth inhibition was assessed at 3-to-5 days depending on the pathogen. To quantify the intensity of inhibition the following scale was used: 0, no inhibition; 1, low inhibition (< 10 mm halus); 2, moderate inhibition (from 10 to < 20 mm), and 3, high inhibition (≥ 20 mm halus). For each *Bacillus* isolate, a global activity index (GAI) was calculated by computing the sum of the scores for the eight plant pathogenic bacteria tested, and the maximum achievable value was 24.

### Statistical analysis

The relationships between the presence of AMP biosynthetic genes, production of cLPs and antibacterial activity in isolates of *Bacillus* from plant environments was determined with Chi-squared distribution with Pearson’s statistic. The frequency of *Bacillus* isolates according to the patterns of detected AMP biosynthetic genes (*ituC*, *bmyB*, *fenD* and *srfAA*) was related to the patterns of cLPs isoforms produced (I1, I2, I3, I4, F1, F2, F3, F4, F5, F6, F7, S1, S2, S3 and S4). In addition, the frequency of isolates according to the degree of antimicrobial activity against the pathogenic bacteria in NA or LB agar (classified as Global Activity Index, GAI low ≤8; GAI high >8 over a maximum of 24) was related to the detected AMP genes and the produced cLP isoforms.

A two-way hierarchical cluster analysis based on Ward's minimum variance criterion was performed [30], which minimizes the total within-cluster variance according to squared Euclidean distance. The analysis clustered *Bacillus* isolates with similar patterns of presence/absence of cLP genes, production of cLP isoforms and antibacterial activity in LB and NA medium. Next, a double hierarchical cluster analysis was performed to reveal which of these clusters showed similar co-occurrence patterns. To find which attributes were responsible of the grouping, a one-way ANOVA was performed.

A correspondence analysis (CA) was performed with the *Bacillus* isolates to obtain relationships between the type of AMP gene (*ituC*, *bmyB*, *fenD* and *srfAA*), low or high antimicrobial activity against the eight plant pathogenic bacteria in NA or LB agar, and the production of cLPs isoforms (I1, I2, I3, I4, F1, F2, F3, F4, F5, F6, F7, S1, S2, S3 and S4). Species and sample origin (aerial plant part or rhizosphere) for each isolate was also introduced in the analysis as an informative variable.

All statistical analysis were carried out using the IBM SPSS Statistics 19.0 package (IBM corporation, USA).

## Results

### *Bacillus* species identification

**Fig 1:**
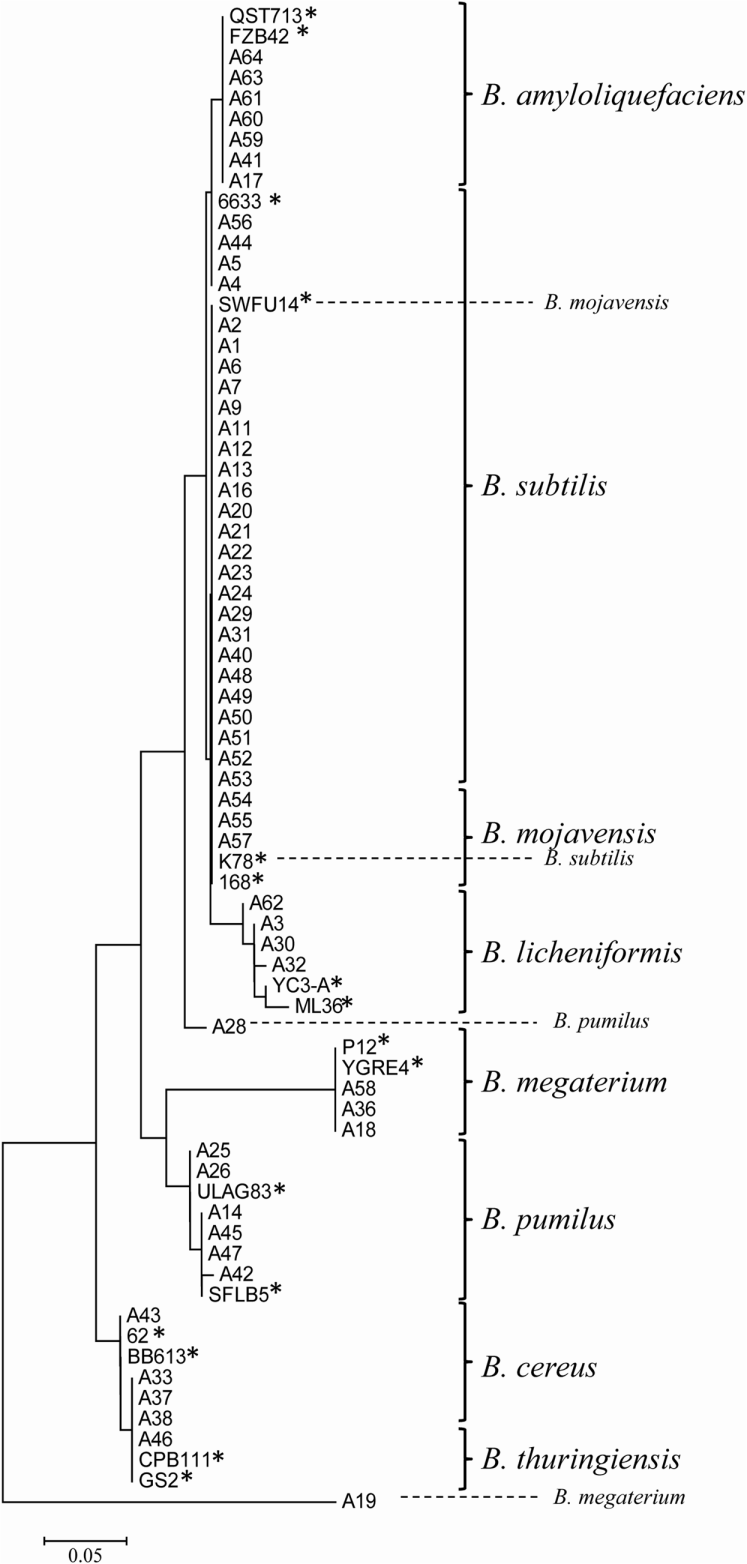
Phylogenetic tree showing interrelationships among *Bacillus* isolates. The tree was done using 73 *16S rDNA* sequences, from 57 *Bacillus* iolates and 16 reference strains (highlighted with an asterisk). The NJ tree was constructed from each data set using the maximum composite likelihood model in MEGA 6.0. The optimal tree with the sum of branch length = 0.57224699 is shown. The tree is drawn to scale, with branch lengths in the same units as those of the evolutionary distances, which were computed using the Poisson correction method, to infer the phylogenetic tree. *Bacillus* were grouped in 8 different species (*B*. *subtilis*, *B*. *amyloliquefaciens*, *B*. *mojavensis*, *B*. *licheniformis*, *B*. *megaterium*, *B*. *pumilus*, *B*. *cereus* and *B*. *thuringiensis*), indicated by means square braquets.

Comparative *16S rDNA* gene sequence analysis of the putative *Bacillus* sub-sample collection was performed to identify them at species level. From the 64 *Bacillus* isolates, a set of 57 were identified as representatives of 8 different species, including 28 *Bacillus subtilis*, 7 *B*. *amyloliquefaciens*, 2 *B*. *mojavensis*, 4 *B*. *licheniformis*, 7 *B*. *pumilus*, 4 *B*. *megaterium*, 4 *B*. *cereus* and 1 *B*. *thuringiensis*. However, 7 isolates were not identified. A NJ tree was performed using 16S rDNA sequences of the *Bacillus* isolates together with 16 reference strains available in the GeneBank. *Bacillus* strains belonging to the identified species were clustered together with the reference species (Fig 1). The phylogenetic analysis revealed eight main clusters of *B*. *subtilis*, *B*. *amyloliquefaciens*, *B*. *mojavensis*, *B*. *pumilus*, *B*. *licheniformis*, *B*. *megaterium*, *B*. *cereus* and *B*. *thuringiensis*. However, the isolates A19 (*B*. *megaterium*) and A28 (*B*. *pumilus*) did not classified into the corresponding main cluster.

### cLPs genes

The results on cLP gene distribution did not differ when comparing the sub-sample of 64 islates with the original collection of 184 isolates previously reported [22]. *bmyB* and *srfAA* genes were the most frequently detected (50 and 48%, respectively), followed by *fenD* (34%), whereas *ituC* was the less frequent gene (21%) A 81.2% of the isolates had at least one of the biosynthetic genes, and a 48.4% had 2-to-4 genes simultaneously.

### cLPs

**Fig 2:**
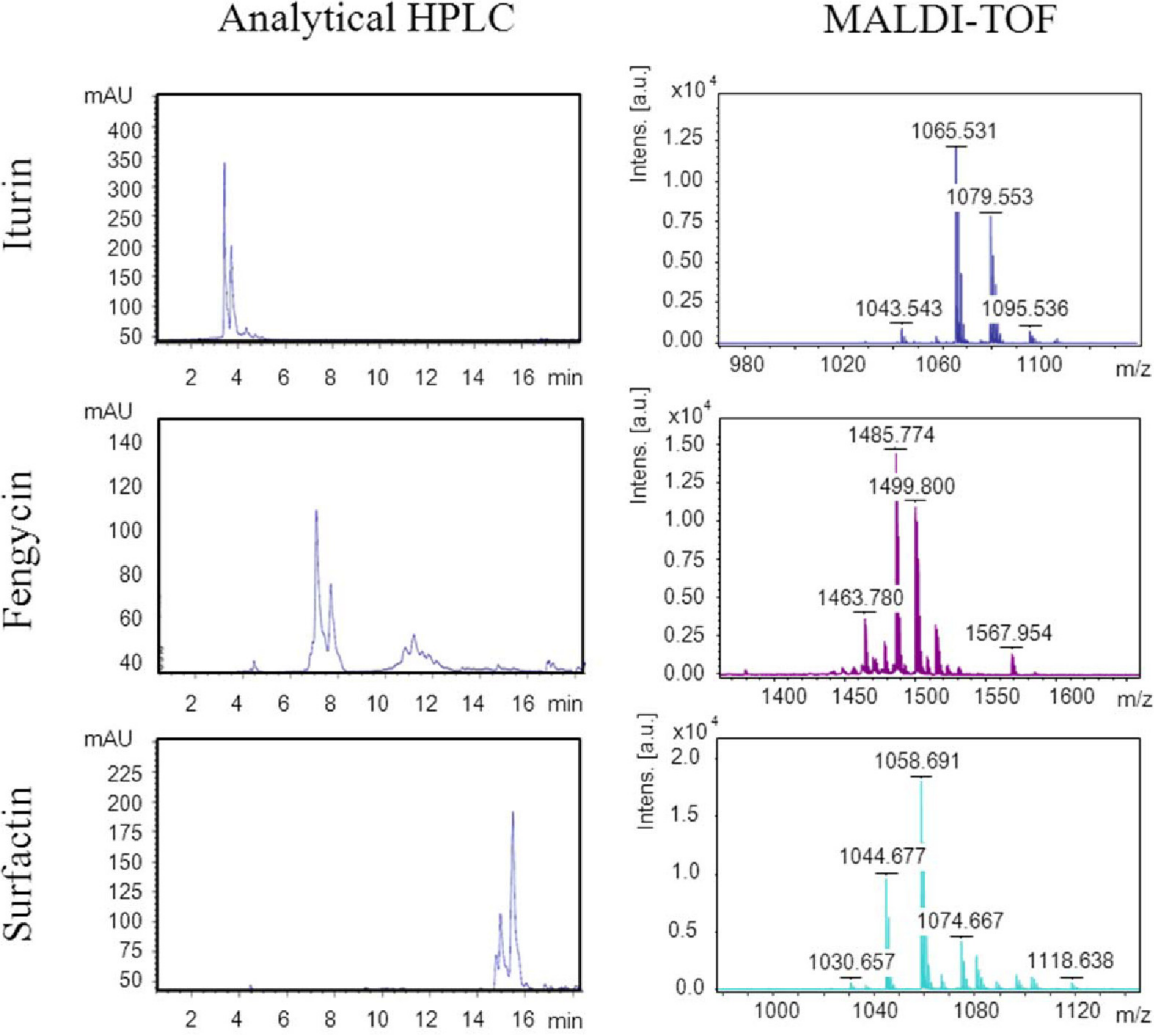
Characterization and identification of cLPs produced by an isolate of *Bacillus amyloliquefaciens*. Analytical HPLC profiles and mass spectra obtained by MALDI-TOF from the parental peaks 1008.7, surfactin, 1065.4, iturin A, and 1464.0, fengycin.

Chromatographic profiles obtained from the organic phases of culture supernatants of *Bacillus* isolates grown in LB broth, showed HPLC differential peaks among strains (3 to 18 min), and common peaks (0 to 3 min), that were associated to the culture medium components. The differential peaks were sampled (a total of 15 samples per isolate), evaporated under vacuum and analyzed by MALDI-TOF and HPLC-ESI-MS. Molecular masses observed were associated to peptides according to the literature [23, 25, 31–34], and the mass spectra were related with surfactins (994–1096 m/z), iturins (1017–1082 m/z) and fengycins (1435–1529 m/z) (Fig 2). Taking into account the results for representative isolates and reference strains, the identity of the molecular mass spectra was confirmed by MALDI-TOF and ESI-MS, and surfactin, iturin, bacillomycin, and fengycin were identified.

Further, the mass spectra of the cLPs were mainly associated with 4 areas of the analytical HPLC chromatography profile (S1 Fig). The first area, between 3 and 5 min was related with the presence of iturin family compounds, including iturin, bacillomycin and mycosubtilin (I1, I2, I3 and I4). Due to the strong biochemical similarities, the differentiation within iturin family components involved a high degree of difficulty. For this reason iturin, bacillomycin and mycosubilin compounds were considered as a unique group, named in this work as iturins. The second and third areas, which included peaks observed from 6 to 9 min and from 11 to 14 min, were related with the presence of compounds of the fengycin family (F1, F2, F3, F4, F5, F6 and F7). Finally, the fourth area was related with surfactin family compounds, which ranged from 14 to 16 min (S1, S2, S3 and S4). Each of these elution peaks was assumed to be a isoform according to the cLPs detected by m/z analysis.

**Fig 3:**
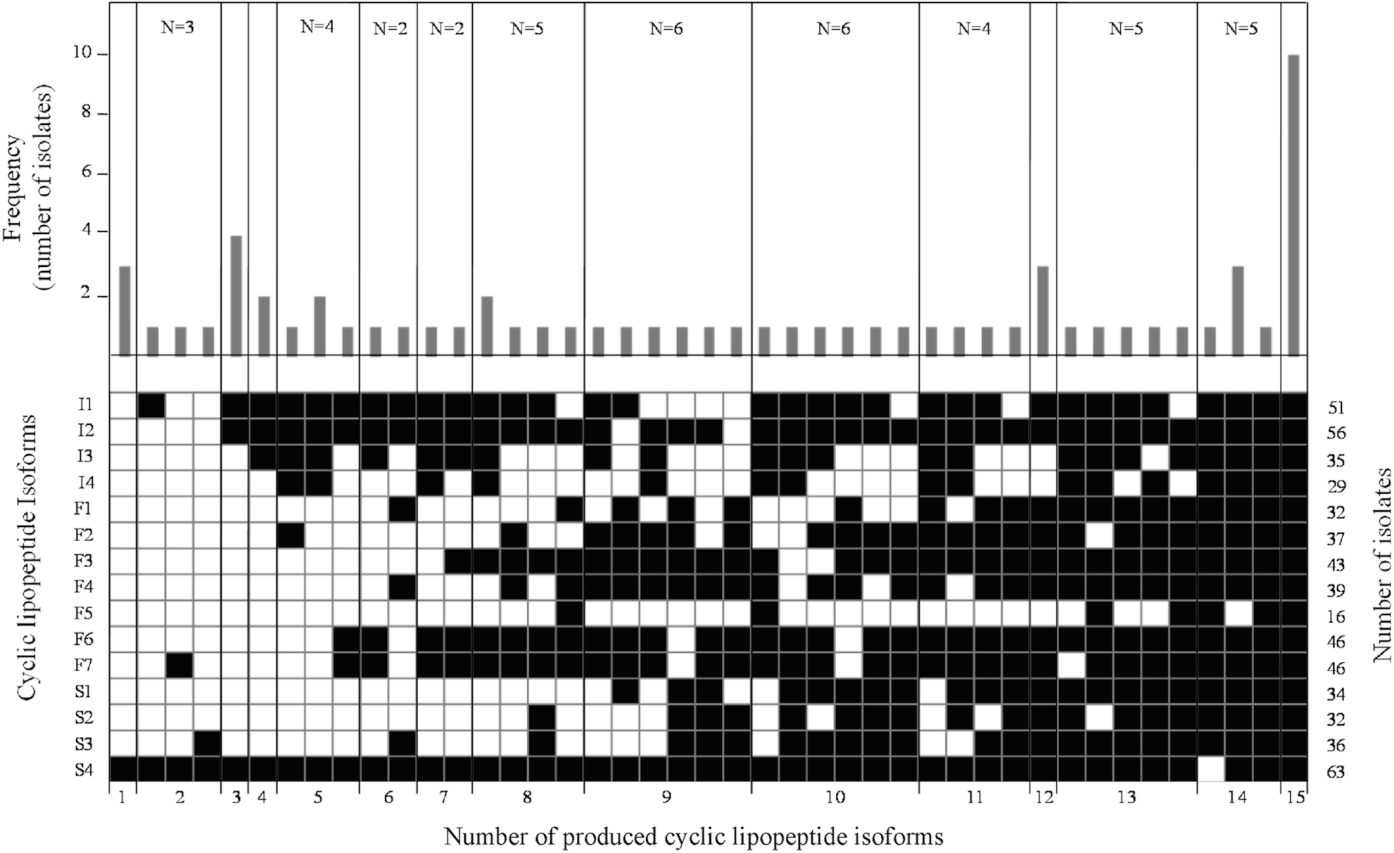
Frequency distribution of cLPs in *Bacillus* isolates from field samples. The number of isolates (N) within each group with simultaneous presence of cLP is indicated in the upper part of the panels. Total number of isolates showing each cLP isoform is shown on the right part of the panel. The presence (■) or absence (□) of cLP is also indicated.

The HPLC profiles of the 64 isolates were determined, providing information of each isolate in relation to the production of cLPs, as well as on the major components produced. The frequency of patterns of cLP isoforms produced was determined, showing a strong heterogeneity among isolates(S2 Fig). Surfactins were the most frequently produced cLPs (98.4% of isolates), followed by iturins (90.6%) and fengycins (79.7%) (Fig 3). Specifically, the most frequently detected isoform was S4, which was detected in 98.4% of isolates, followed by I1 and I2 (79.7% and 87.5% of isolates, respectively), and F6 and F7 (both detected in 71.9% of isolates). While the less frequent isoform was F5, detected in 25% of isolates. The simultaneous production of iturins, fengycins and surfactins was observed in 75% of isolates.

We observed a high diversity and a wide range of situations among isolates (number and the quantity of isoforms produced), revealing a strain specific pattern, generally associated to the *Bacillus* species. For example, *B*. *amyloliquefaciens* and *B*. *mojavensis* isolates produced all isoforms, except the reference strain *B*. *amyloliquefaciens* QST713 that did not produce F6, F7 and S2 isoforms. The production patterns of *B*. *subtilis* were more heterogeneous. The production of all cLPs isoforms was detected only in one of the isolates, although I3, I4, and F5 were less frequent in comparison to *B*. *amyloliquefaciens* and *B*. *mojavensis*. *B*. *licheniformis* and *B*. *megaterium* patterns were also heterogeneous and distinguished by the lack of surfactins. In contrast, *B*. *pumilus* isolates showed mainly surfactins, as well as, the isoforms I1, I2, F6 and F7. Finally, *B*. *cereus* and *B*. *thuringiensis* patterns were characterized by a lack of fengycins and surfactins.

### Antibacterial activity

**Fig 4:**
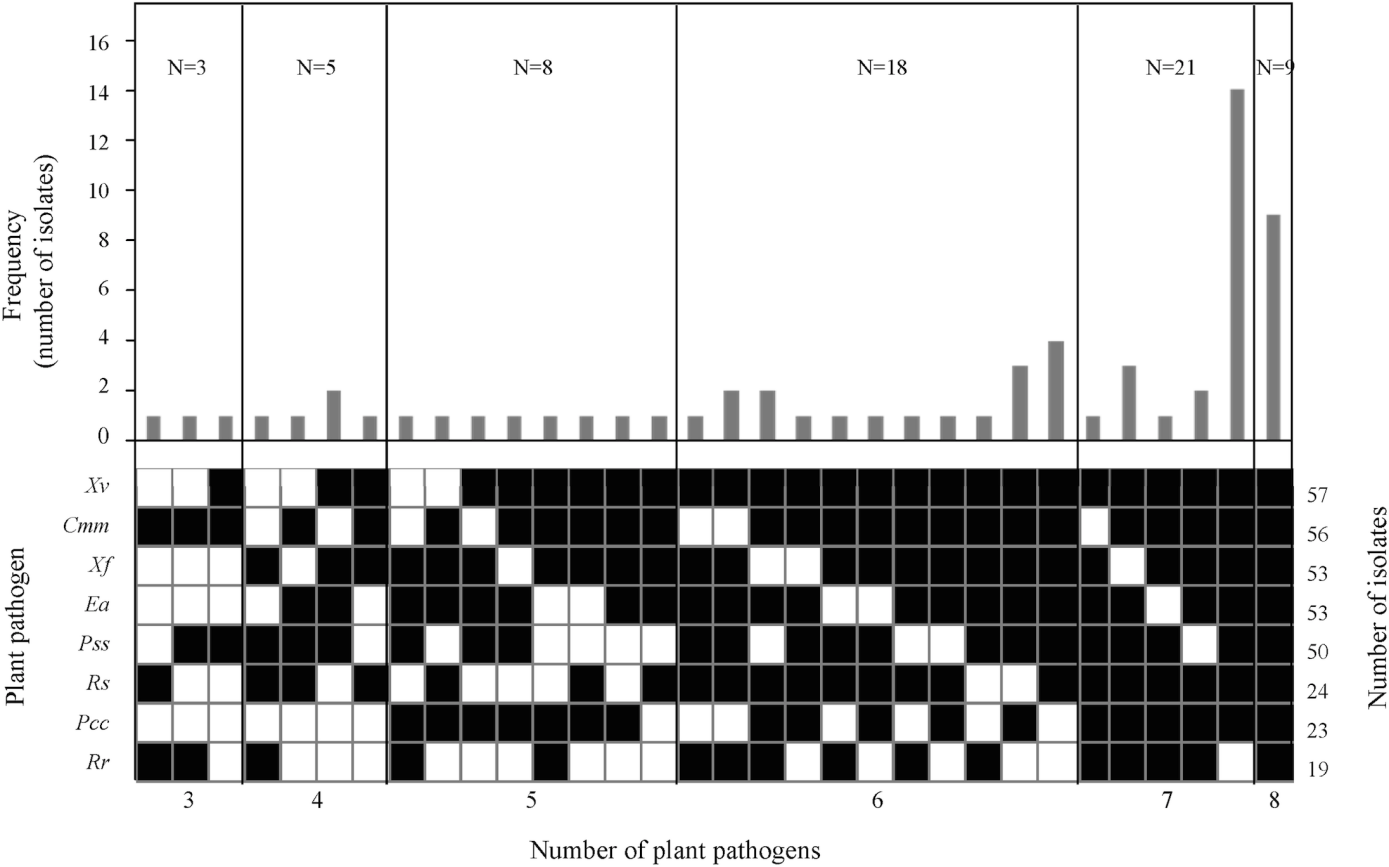
Frequency distribution of antibacterial activity in *Bacillus* isolates from field samples. The number of isolates (N) within each group with antibacterial activity in LB and NA growth media against the same number of bacterial plant pathogens is indicated in the upper part of the panels. Total number of active isolates per pathogen is shown on the right part of the panel. The presence (■) or absence (□) of activity is also indicated.

Most isolates showed antagonism very rapidly after 24 h against the bacterial plant pathogens in the two growth media. In LB agar, 89.1% of *Bacillus* showed an inhibitory effect against at least one of the plant pathogenic bacteria, and a 28.1% were highly inhibitory (global antibacterial activity >8 over a maximum of 24). In NA agar, all strains showed inhibitory effects and 56.2% were highly inhibitory. There was a differential activity between the isolates depending on the plant pathogenic bacteria targeted and growth medium, and an increase of the number of antagonistic isolates in NA was observed in comparison to LB agar. More in detail, an increase of the number of active strains against *E*. *amylovora* and *P*. *syringae* and a decrease of the number of active strains against *X*. *arboricola* was observed in NA compared to LB. In contrast, the number of active strains against *P*. *carotovorum*, *R*. *solanacearum X*. *axonopodis*, *R*. *radiobacter* and *C*. *michiganensis* was similar in both growth media. Globally, 75% of isolates showed a strong antibacterial activity, which inhibited the growth of 6 to 8 bacterial plant pathogens (Fig 4).

### cLP genes and products

cLPs produced by the *Bacillus* isolates according to the HPLC profile (individual peak detection and grouping peaks in families of cLPs), were related to the presence of the cLP genes in each strain, using Pearson’s R correlation values obtained by the Chi squared test. Genes *bmyB* and *ituC* were related with the production of iturins (as the sum of iturin, bacillomycin and mycosubilin). The production of iturins, fengycins and surfactins was detected in all isolates containing the genes *ituC*, *fenD* and *srfAA*, respectively. More specifically, of the 64 *Bacillus* isolates, 31 that had the *srfAA* gene produced surfactins, whereas 33 isolates that produced surfactin did not show the *srfAA* gene. In the same way, 22 isolates that had the *fenD* gene produced fengycins, but 29 that did not show the *fenD* gene produced fengycins. Also, 39 isolates that showed *ituC* and/or *bmyB* genes produced iturins, whereas 4 isolates that had *ituC* genes did not produce iturins, and 23 isolates that did not have *bmyB* genes produced iturins. Globally, the presence of the *fenD* gene was significantly related to the production of six fengycin isoforms (F1, F2, F3, F4, F6 and F7) (R = 0.365, p<0.05). In contrast, the presence of the *ituC* gene was only related to the production of one iturin isoform (I1) (R = 0.267, p<0.05). No significant relationships were observed either between the presence of *bmyB* and the production of iturins, or the presence of the *srfAA* gene and the production of surfactins.

### Antibacterial activity and cLP genes

Antibacterial activity was related to the presence of cLP genes. According to the Chi squared test, strong antibacterial activities in isolates in LB medium were significantly related with the presence of *srfAA* and *fenD* genes (R = 0.645, p<0.05; R = 0.572, p<0.05, respectively). In more detail, the high antibacterial activity of *Bacillus* isolates against *R*. *solanacearum*, *R*. *radiobacter*, *E*. *amylovora*, *P*. *syringae* pv. syringae and *X*. *axonopodis* pv. vesicatoria, was related with the presence of *srfAA* and *fenD* genes; against *C*. *michiganensis* sbsp. *michiganensis*, with the *fenD* gene, and against *P*. *carotovorum* sbsp. *carotovorum*, with the *srfAA* gene. In addition, only the activity against *R*. *radiobacter* was significantly related with the presence of *bmyB* gene. In contrast, the antimicrobial activity of isolates in NA was not related to the presence of either *ituC*, *bmyB*, *fenD or srfAA* genes. Taking into account both growth media and all bacterial pathogens, there was a significant relationship between strong antibacterial activity and the presence of *srfAA* gene (R = 0.307, p<0.05).

### Antibacterial activity and cLP production

All the isolates active against the bacterial plant pathogens in LB and up to 85% in NA produced at least one type of cLP. A significant relationship between the production of iturin (I3, I4) and fengycin (F2, F3, F5, F6, F7) with the high antibacterial activities in LB agar was observed (R = 0.412; 0.448; 0.379; 0.412; 0.413; 0.419; 0.419, respectively, all of them p<0.05). Strong antibacterial activity in NA was significantly related with the production of fengycin (F1, F2, F3, F4; R = 0.447; 0.407; 0.349; 0.415, respectively, p<0.05), and surfactin (S1, S2,S3; R = 0.316; 0.358; 0.324, respectively, p<0.05). More in detail, the high activity against *R*. *solanacearum* was related with the production of most cLP isoforms (I2, I3, I4, F1, F2, F3, F4, F5, F6, F7, S1, S2 and S3). In contrast, only the production of F2 was significantly related with the high antibacterial activity in NA against 5 out of 8 plant pathogens. Taking into account both growth media, there was a significant relationship between the strong antibacterial activity and the production of fengycin (F1, F2, F3, F4) and surfactin (S1, S2, S3).

### Relationships between antibacterial activity, cLP genes and products

To study triple relationships, a binary array was performed using the 21 attributes as variables for each isolate. These attributes were the production of cLPs according to the HPLC profile (I1, I2, I3, I4, F1, F2, F3, F4, F5, F6, F7, S1, S2, S3 and S4), the presence of cLP genes (*ituC*, *bmyB*, *fenD* and *srfAA*), and the global inhibitory activity against bacterial plant pathogens (in LB and NA media).

**Fig 5:**
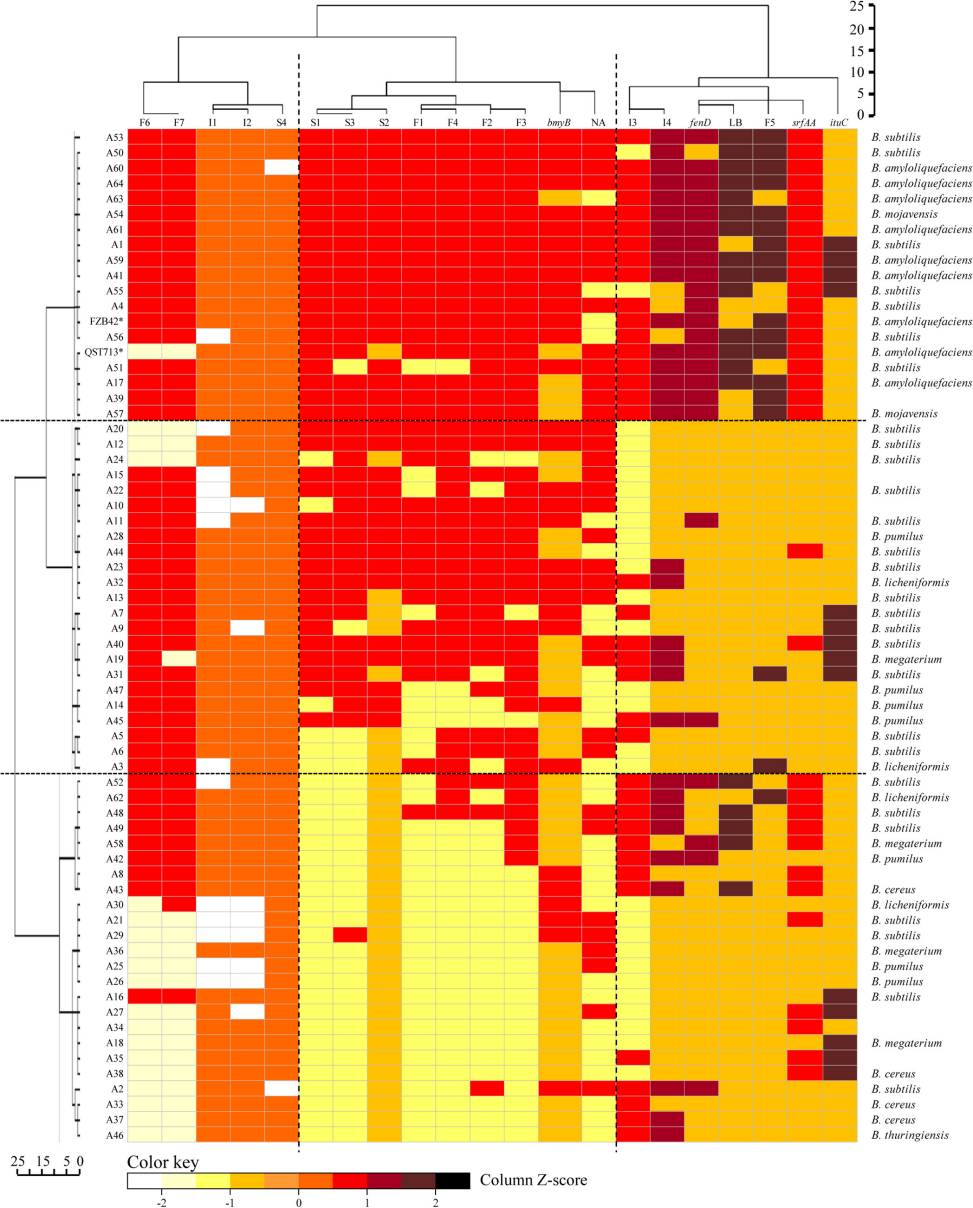
Two-way hierarchical clustering of *Bacillus* isolates. The analysis was performed using as attributes the production of cLPs grouped by the three families of (iturins (I), fengycins (F) and surfactins (S), the presence of four cLP biosynthetic genes (*srfAA*, *fenD*, *bmyB*, and *ituC*), and antibacterial activity in LB and NA growth media. Strains QST713 and FZB42 are included as reference strains. The similarity matrix was calculated using the squared euclidean distance at the discretion of Ward’s. Cluster analysis was performed by the Agglomerative Hierarchical Group Method through the cluster analysis procedure of IBM-SPSS package. The colours correspond to the Z-scores of each attribute for a given isolate. The higher (or lower) the Z-score, the stronger the intensity of the clustering. A Z-score near zero indicates no apparent spatial clustering.

The two-way cluster analysis showed a cluster for isolates and a cluster for attributes (Fig 5). The first cluster showed three groups of isolates (at 10 units distance), each group represented by significant attributes. In the same way, the second cluster showed three groups of variables each represented by significant *Bacillus* species. The ANOVA analysis indicated that the variables with a significant contribution in the cluster formation were *srfAA* gene, surfactins (S1, S2 and S3), *fenD* gene, fengycins (F1, F2 F3, F4, F5, F6 and F7), iturins (I3 and I4), and antibacterial activity in LB and NA media (F values ranging from 5.159 to 44.425 and p-values <0.05). However, some variables, such as production of I1, I2 and S4 and the presence of *bmyB* and *ituC* genes were not significant to determine the grouping of isolates (F values ranging from 0.003 to 2.467 and p-values >0.05). The Tukey post *hoc-test* revealed that the variable *srfAA* gene distinguished the three clusters through their cluster means. Moreover, the variables I3, I4, F5, *fenD* gene and antibacterial activity in LB medium differentiated the group A from the groups B and C, and the variables F1, F2, F3, F4, S1, S2, S3, *bmyB* gene and antibacterial activity in NA medium differentiated groups A and B from C (Fig 5).

The greatest differences were found between groups A and C, whereas group B was intermediate. Thus, group A included strong antibacterial isolates that produced most cLPs, and with several cLP genes simultaneously (most strains with 3 or more). Group B was characterized by intermediate isolates with lower antibacterial activity than group A, non-active in LB, but a high frequency of isolates active in NA medium. Although simultaneous cLP genes was still present in group B, the frequency was lower than in group A. Group C consisted of isolates with low antibacterial activity, especially in LB agar, producing few cLPs and with a low number of cLP genes simultaneously (most strains with 2 or less cLP genes). More in detail, group A isolates produced F1, F2, F3, F4, F6, F7, S1, S2 and S3, and remarkably I3, I4 and F5, contained *bmyB*, *srfAA* and *fenD* genes simultaneously, and showed high antibacterial activity in LB and NA media, including *B*. *subtilis*, *B*. *amyloliquefaciens* and *B*. *mojavensis*, and the last two were exclusive to this group. Group B was mainly composed of isolates that produce F2, F3, F4, F6, F7, S1 and S3, absence of *sfrAA* and *fenD* genes, and showed no antibacterial activity in LB medium, including *B*. *subilits*, *B*. *licheniformis*, *B*. *megaterium*, and *B*. *pumilus*. Finally, group C consisted of isolates with low production or absence of F1, F2, F3, F4, F5, S1, S2 and S3, low presence of *bmyB* and *fenD* genes, and low antibacterial activity in LB agar (Fig 5) and was the most heterogenous group, that included *B*. *subilits*, *B*. *licheniformis*, *B*. *megaterium*, *B*. *pumilus*, *B*. *cereus* and *B*. *thuringiensis*, although the last two were exclusive to this group.

**Fig 6:**
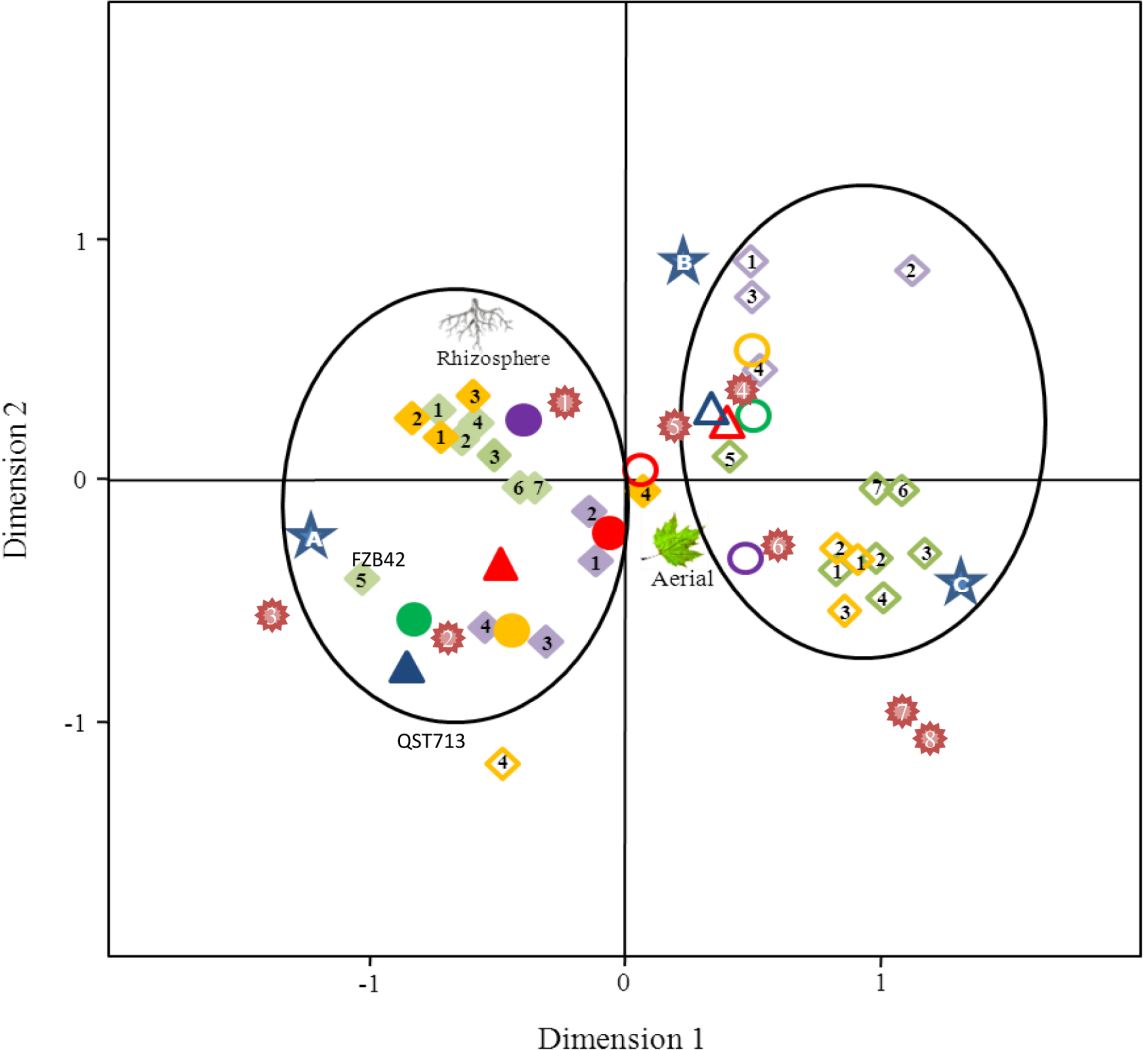
Map of the correspondence analysis of *Bacillus* isolates. The analysis was performed according to the presence of cLP biosynthetic genes (*srfAA*, yellow **○**; *fenD*, green **○**; *bmyB*, purple **○**; or *ituC*, red **○**), production of cLPs related to iturin, fengycin or surfactin families I1, I2, I3, I4 (violet ◊), F1, F2, F3, F4, F5, F6, F7 (green ◊), S1, S2, S3 or S4 (yellow ◊) and antibacterial activity in LB (blue △) or NA (red △) media (GAI low or high). Supplementary information of the Species grouping in the phylogenetic analysis (✹; *B*. *subtilis* (1), *B*. *amyloliquefaciens* (2), *B*. *mojavensis* (3), *B*. *pumilus* (4), *B*. *licheniformis* (5), *B*. *megaterium* (6), *B*. *cereus* (7) and *B*. *thuringiensis*(8)) and sample origin (aerial plant part or rhizosphere) has been added. Positive scores (filled symbols) and negative scores (empty symbols) are indicated. The two main groups of variable scores defined by the dimensions are highlighted. The three groups of strains obtained in the hierarchical analysis are also indicated with a star.

The correspondence analysis performed using the above mentioned variables also confirmed two main groups of variable scores defined by the two dimensions, explaining a 48.8% of the variability (Fig 6). The first dimension was represented by the cLP products, especifically surfactins S1 (0.667) and S2 (0.636), and fengycins F1 (0.614), F2 (0.646), F3 (0.698) and F4 (0.645). The second dimension was mainly described by the cLP products related with the iturin family I3 (0.519) and I4 (0.324), and the biosynthetic gene *srfAA* (0.334). Interestingly, the variables were divided into two groups. The first group (left part of the panel) was represented by the presence of a high number of simultaneous cLP genes per *Bacillus* strain, presence of *bmyB*, *srfAA* and *fenD* genes, and production of cLPs related with iturin, fengycin and surfactin cLP families (I1, I2, I3, I4, F1, F2, F3, F4, F5, F6, F7, S1, S2, S3 and S4), and a high antibacterial activity in both LB and NA growth media. The first group (left panel) was defined by the species *B*. *amyloliquefaciens*, *B*. *mojavensis* and *B*. *subtilis* (including the commercial strains QST713 and FZB42). The second group (right part of the panel), was represented by a low number of simultaneous cLP genes per strain, absence of *bmyB*, *srfAA* and *fenD* genes, low production of cLPs related with iturin, fengycin and surfactin families, low antibacterial activity and defined by the species *B*. *pumilus*, *B*. *licheniformis*, *B*. *megaterium*, *B*. *cereus* and *B*. *thuringiensis*.

The three groups of isolates described previously by the hierarchical two-way cluster analysis were associated to different areas of the map of the correspondence analysis (Fig 6). Thus, group A was located in the lower left panel, characterized by isolates with *fenD* and *srfAA* genes, active against bacterial plant pathogens in both growth media, mainly iturin producers and isolated from rhizosphere samples. Group B was located in the upper right panel, characterized by isolates with *bmyB* genes, fengycin and surfactin producers and isolated from both rhizosphere and aerial plant parts. Group C was located in lower right panel, mainly composed of isolates without *bmyB* gene, not active against bacterial plant pathogens, which did not produce of fengycins and surfactins and isolated from aerial plant parts.

## Discussion

Several members of the genus *Bacillus*, mainly *B*. *subtilis/amyloliquefaciens*, have been the object of studies for the identification of biosynthetic genes involved in antimicrobial activity, to establish the role of their expression products in biological control of plant pathogens [18, 20, 23, 35–40]. However, there are no reports searching for relationships between the presence of biosynthetic genes and their corresponding products, with the antagonistic activity against bacterial plant pathogens. In the present study, a collection of *Bacillus* isolates obtained from plant environments [22] was characterized and we were able to find relationships between antibacterial activity against plant pathogenic bacteria, and cLP biosynthetic genes and products.

A detailed analysis of the 16S rDNA sequences in the isolates revealed that the collection was composed of eight different *Bacillus* species, including *B*. *subtilis*, *B*. *amyloliquefaciens*, *B*. *pumilus*, *B*. *licheniformis*, *B*. *mojavensis B*. *megaterium*, *B*. *cereus*, and *B*. *thuringiensis*. Interestingly, most of our isolates pertained to the *B*. *subtilis* group, in agreement with other reports in which among the genus *Bacillus*, *B*. *subtilis* was the most frequently isolated [41,42]. Also, we have found a wide diversity within the isolates from plant environments, covering most of the *Bacillus* species reported in the litterature. Currently, *Bacillus* species are included in two specific groups; the first one refers to the *Bacillus subtilis* group that includes *B*. *subtilis*, *B*. *pumilus*, *B*. *licheniformis*, *B*. *mojavensis*, *B*. *megaterium* and *B*. *amyloliquefaciens*, and the second group refers to the *Bacillus cereus* group, that is composed of *B*. *cereus*, *B thuringiensis*, *B*. *mycoides* and *B*. *anthracis*. These grouping has been proposed based on a set of combination of molecular and biochemical methods [43, 44] restriction enzyme DNA patterns [45], mas spectrometry by MALDI-TOF [46], and *16S rDNA*, *rpoB* and *gyrP* gene sequencing [44, 45, 47–49].

We showed that the cLPs genes, *bmyB*, *srfAA* and *fenD* were the most frequently detected among isolates of *Bacillus* associated to plants, primarily in *B*. *amyloliquefaciens*, followed by *B*. *mojavensis* and *B*. *subtilis* species. In addition, the number of cLP genes per strain tends to be multiple, in most cases 2 to 4 simultaneously. This finding is in agreement with the widespread presence of these genes in the genomes of known strains reported as biocontrol agents of plant diseases or plant growth promoters or the existence of several operons involved in antimicrobial peptide biosynthesis accounting for up to 10% of the genome [13, 24, 25]. Also, the dominance of these particular genes among the natural population of *Bacillus* associated to plants reinforces the evidence of a competitive role of bacillomycin, surfactin and fengycin which have been argued to be implicated in the fitness of these bacteria in natural environments [9, 50, 51].

The production of cLPs among the isolates showed differential patterns in isoforms within the main compounds (iturins, fengycins, surfactins), that revealed species and strain specific patterns. Most of *B*. *amyloliquefaciens* and *B*. *mojavensis* isolates produced all the isoforms (with the exception of QST713), but the pattern was more heterogeneous in the other *Bacillus* species. This is in agreement with several reports studying the production of cLPs in *B*. *subtilis* and *B*. *amyloliquefaciens* [2,3,9,11,13, 20, 25, 34, 35, 52–54], although the production of iturins, surfactins and fengycins has been reported in *B*. *mycoides* [18] and even in *B*. *thuringiensis* [55]. Interestingly, surfactins were the most abundantly produced cLPs in our isolates, being C15 the most frequent homologue, in agreement with Malfanova et al. [52]. Besides, most of our isolates produced more than one cLP and isoforms simultaneously, in agreement with other studies restricted to particular strains, like the production of fengycin and iturin simultaneously by *B*. *subtilis* UMAF6639 [23], of iturin A, fengycin and surfactin A simultaneously by *B*. *subtilis* CMB32 [53], or of surfactins and iturins by *B*. *subtilis* K1 [56].

Most of the *Bacillus* isolates showed antibacterial activity and inhibited at least 1 out of 8 plant pathogenic bacteria in the two growth media used (LB and NA), and 75% of the isolates inhibited six or more. Our results confirm that the more active isolates were *B*. *amyloliquefaciens*, while the less active were the isolates included in the group of *B*. *cereus*. Unfortunatelly, most reports have focused on antifungal activity of *Bacillus* species and against a restricted group of fungal plant pathogens [28, 54, 57–62]. When the antagonistic activity of *Bacillus* has been studied against bacterial plant pathogens, it was frequently focused on a specific strain and to control a single target bacterial plant pathogen [4, 63]. There is only a report of a collection of *Bacillus* isolates that were antagonists to a broad range of phytopathogenic bacteria [19].

The relationship between cLP genes and products was significant for the *fenD* gene and the production of fengycins (both individually or as a group of products). More specifically, the sensitivity of primers directed to the *fenD* gene was high enough and rather consistent among strains of *Bacillus* [22], in agreement with the fact that *fenD* sequences obtained from the GenBank showed a high homology at inter and intra species level (data not shown). The presence of the *ituC* gene was related to the production of only one iturin isoform, and this was not expected. In this case the *ituC* primers had high sensitivity, but some limitations of the PCR would be attributed to the heterogeneity of the gene sequences among our population of isolates. However, the relationship between genes and products was not significant between *bmyB* or *srfAA* and the corresponding gene products.

The lack of relationship between genes and products can be explained by a fail to detect the genes by PCR or to a lack of production of the cLPs in some isolates. The limitations in terms of sensitivity of PCR may be due to the intrinsic diversity at genetic level among our collection of *Bacillus*, which may limit the capacity of a primer set to detect all the variations of a specific gene. This was evident in cases of isolates which showed production of cLPs without apparently harbouring the corresponding genes, that resulted in a non-significant relationship. In these cases, cLP gene detection could be improved using another target from the same cluster. In addition, some strains having certain cLP genes might not be producing the expected products in the growth media used in the present work. This is in agreement with reports describing that the presence of antibiotic biosynthetic operons is not always associated with the production of the gene product. This fact could be due mainly to gene mutation as it has been described in *Bacillus* strain 168 that fails to produce surfactin and fengycin due to a frameshift mutation in *sfp* encoding a transferase that is required to activate the corresponding synthases [64,65]. Also, a differential production of cLPs in function of the growth conditions have been reported [66].

Studies focused on cLP production and the antibacterial properties of strains of *Bacillus* are scarce, and only some evidences have been provided on the antibacterial activity of iturin, surfactin and fengycin [24,25]. For example, it has been reported the activity of surfactin in the reduction of infections by *P*. *syringae* in *Arabidopsis in vitro* plants [63] or the role of iturin in the inhibition of infections caused by *X*. *campestris* pv. cucurbitae and *P*. *carotovorum* subsp. *carotovorum* in detached melon leaves [11]. Other studies have described that surfactins and iturins may be, in addition to antimicrobial activity, involved in the capacity to colonize plant surfaces or to form biofilms [35,51].

Two consistent and well defined groups of *Bacillus* isolates were revealed using cluster and correspondence statistical procedures with the three characteristics (presence of cLP biosynthetic genes, production of cLPs and antibacterial activity). The first group included isolates characterized by having several cLP genes simultaneously (specifically the pattern *bmyB*-*fenD*-*srfAA*), the production of a wide range of iturins, fengycins and surfactins, and the intense and wide spectrum of antibacterial activity represented by the species *B*. *amyloliquefaciens*, *B*. *mojavensis* and *B*. *subtilis*. When the first group was analyzed in more detail, it was observed that a close cluster of isolates have been obtained from rhizosphere samples (data not shown). In addition, the reference strains *B*. *amyloliquefaciens* QST713 and FZB42, which are reported as efficient biocontrol agents, were, as expected, included in this group. The second clearly distinguishable group was composed of isolates with a low number of cLP genes simultaneously, poor production of cLPs, and low antimicrobial activity defined by the species *B*. *pumilus*, *B*. *licheniformis*, *B*. *megaterium*, *B*. *cereus* and *B*. *thuringiensis*. The most relevant features for the differentiation of both groups were the presence of *fenD* gene, the production of fengycins and iturins, and the antibacterial activity in LB agar. The high frequency of detection of cLP genes in the first group was in agreement with the widespread presence of these genes in the genomes of known strains of *B*. *subtilis* and related species which have been reported as biological control agents [13,18,20,25]. Interestingly, most commercial microbial biofungicides with *Bacillus* strains as active ingredients are *B*. *amyloliquefaciens/subtilis*, that reinforces our findings [67]

Finally, there is a strong demand of new strains of microorganisms to provide active ingredients for the rapidly growing field of microbial biopesticides [67]. The main mechanisms of plant disease control by these microorganisms are very diverse and frequently rely on antibiosis, colonization and competition for resources and entry sites with the pathogen, and elicitation of defense responses on host [1,3,5,8,63, 67, 68]. Unfortunately, in view of the diversity of mechanisms of action the chance of selecting effective biological control strains during the screening of large collections of isolates is usually very low and requires considerable effort [67]. This is probably the reason for that antibiosis is often prefered as the target mechanism of selection and because most commercial microbial pesticides exhibit antibiosis as the main mechanism of action. Then, according to our results, the selection of *Bacillus* strains using a combination of differential genetic and phenotypic traits (e.g. multiple cLP genes and production of several cLPs simultaneously, wide spectrum of antagonism), is expected to increase the chances of finding potential biocontrol agents of plant diseases.

## Supporting Information

S1 Fig Scheme of a typical HPLC profile. Illustration of the 15 peaks related with the presence of iturin (I), fengycin (F) and surfactin (S) cyclic lipopeptides, grouped in three clusters according to the corresponding family.
(TIFF)

S2 Fig HPLC profiles of *Bacillus* isolates. HPLC profiles of organic extracts from cultures of 10 representative *Bacillus* isolates.
(TIFF)
