# Supplementary figures and images for: Cyclic Lipopeptide Biosynthetic Genes and Products, and Inhibitory Activity of Plant-Associated Bacillus against Phytopathogenic Bacteria
Source: PLoS One. 2015 May 29;10(5):e0127738. doi: 10.1371/journal.pone.0127738 (PMC4449161; doi:10.1371/journal.pone.0127738)

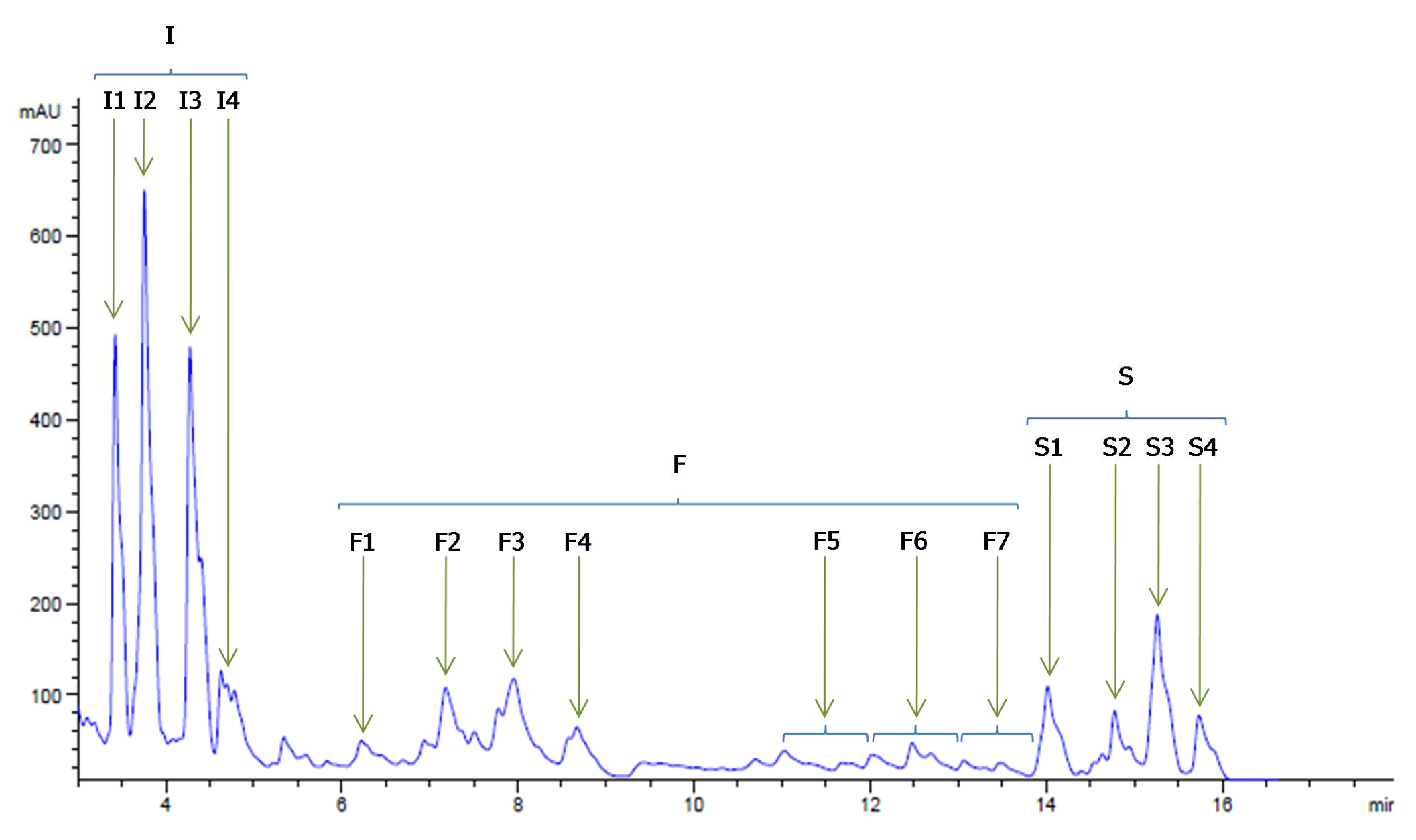

Supplement: S1 Fig — Illustration of the 15 peaks related with the presence of iturin (I), fengycin (F) and surfactin (S) cyclic lipopeptides, grouped in three clusters according to the corresponding family. (TIFF) [file pone.0127738.s001.TIFF]

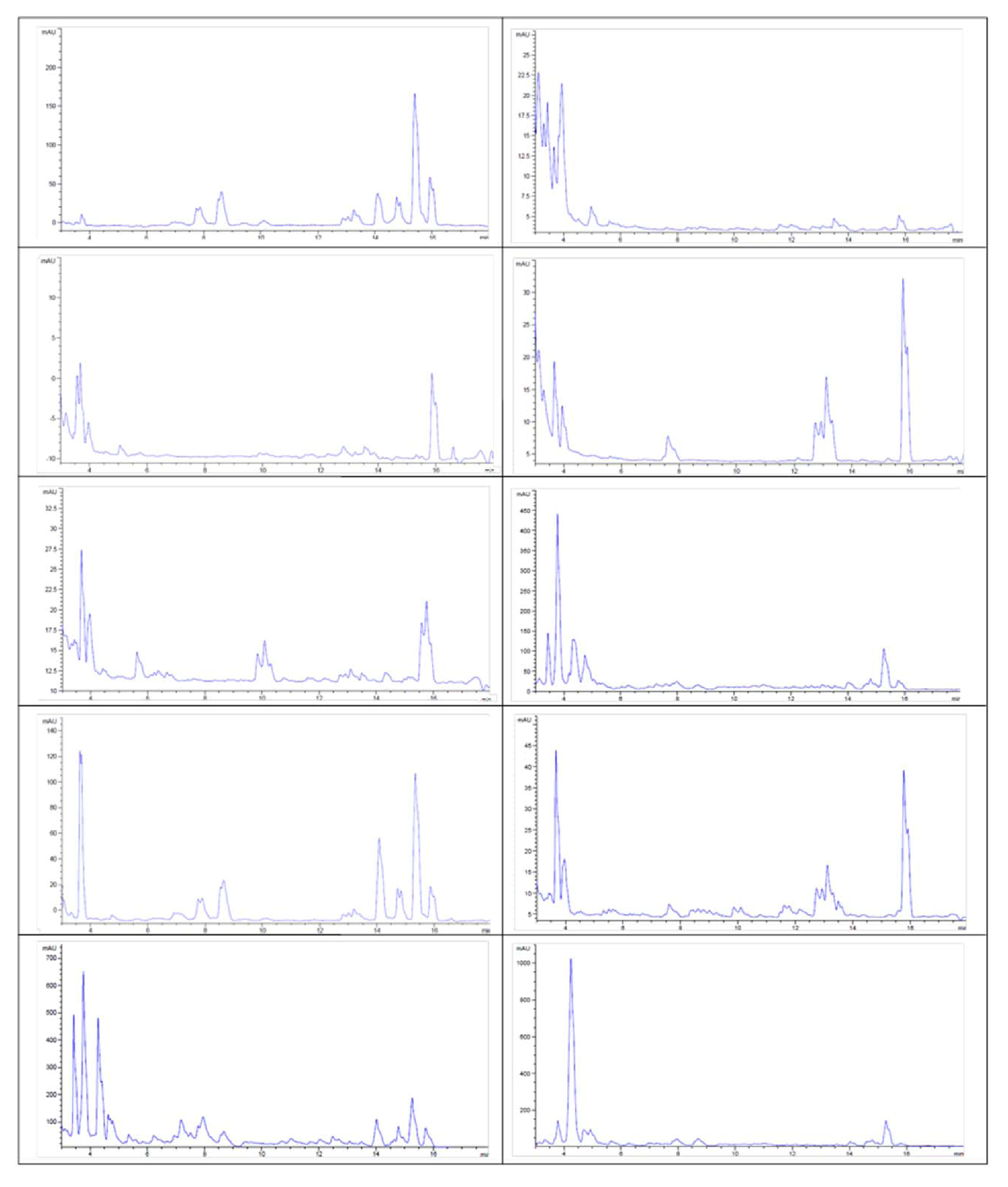

Supplement: S2 Fig — HPLC profiles of organic extracts from cultures of 10 representative Bacillus isolates. (TIFF) [file pone.0127738.s002.TIFF]
